# Early-onset neonatal sepsis: Organism patterns between 2009 and 2014

**DOI:** 10.1093/pch/pxz073

**Published:** 2019-08-09

**Authors:** Michael Sgro, Douglas M Campbell, Kaitlyn L Mellor, Kathleen Hollamby, Jaya Bodani, Prakesh S Shah

**Affiliations:** 1 Keenan Research Centre of the Li Ka Shing Knowledge Institute, St Michael’s Hospital, Toronto, Ontario; 2 Department of Pediatrics, St. Michael’s Hospital, Toronto, Ontario; 3 Centre for Urban Health Solutions, Li Ka Shing Knowledge Institute, St. Michael’s Hospital, Toronto, Ontario; 4 Division of Neonatology, Department of Pediatrics, University of Toronto, Toronto, Ontario; 5 Faculty of Medicine, University of Toronto, Toronto, Ontario; 6 Department of Pediatrics, Regina Qu’Appelle Health Region, Regina, Saskatchewan; 7 Department of Pediatrics, Mount Sinai Hospital, Toronto, Ontario; 8 Maternal-Infant Care Research Center, Mount Sinai Hospital, Toronto, Ontario

**Keywords:** E coli, Group B streptococcus (GBS), Infant, NICU

## Abstract

**Objective:**

To evaluate trends in organisms causing early-onset neonatal sepsis (EONS). Congruent with recent reports, we hypothesized there would be an increase in EONS caused by *Escherichia coli.*

**Study Design:**

National data on infants admitted to neonatal intensive care units from 2009 to 2014 were compared to previously reported data from 2003 to 2008. We report 430 cases of EONS from 2009 to 2014. Bivariate analyses were used to analyze the distribution of causative organisms over time and differences by gestational age. Linear regression was used to estimate trends in causative organisms.

**Results:**

Since 2003, there has been a trend of increasing numbers of cases caused by *E coli* (P<0.01). The predominant organism was *E coli* in preterm infants and Group B Streptococcus in term infants.

**Conclusions:**

With the majority of EONS cases now caused by *E coli*, our findings emphasize the importance of continued surveillance of causative organism patterns and developing approaches to reduce cases caused by *E coli*.

Early-onset neonatal sepsis (EONS) remains a significant cause of neonatal morbidity and mortality (1). Estimates of the incidence of EONS range from 0.54 to 1.19/1,000 live births (2–5) or 9 to 12/1,000 neonatal intensive care unit (NICU) admissions (6,7). EONS is largely caused by vertical transmission of bacteria and has been defined by a clinical presentation at <2 to 7 days after birth (2–20).

Group B Streptococcus (GBS) has historically been implicated as the most important organism causing EONS (21–23). Prevention efforts have focused on vertical transmission of GBS through maternal intrapartum antibiotic prophylaxis (IAP) (21,22,24). Recent reports have demonstrated a changing epidemiology of EONS, with a reduction in cases caused by GBS and a relative increase in cases caused by *Escherichia coli* (12,13). Other studies have shown an absolute increase in EONS caused by *E coli* (3). It remains unclear if the incidence of *E coli* EONS is truly increasing due to factors such as IAP (17), or if it is attributable to advancements in neonatal resuscitation that have resulted in greater survival of preterm infants born at increasingly younger gestational ages (13,25–29) and in whom *E coli* is the most common organism (1,3,14). There have also been several studies showing *E coli* antibiotic resistance in infants including a Canadian study (30).

There is an ongoing need to understand the current EONS epidemiology to ensure appropriate IAP and empiric treatment for suspected EONS. Our group previously analyzed EONS in infants admitted to Canadian NICUs from 2003 to 2008 and reported a significant decrease in GBS (31). Our group also reported that GBS was more common in term infants and *E coli* was more common in preterm infants. The aim of this current study was to longitudinally investigate the incidence of and pathogens causing EONS, thus furthering our understanding of the changing epidemiology. In line with recent findings from the USA (3,12–14,16), we hypothesized there would be a trend of increased EONS caused by *E coli* that was most pronounced in preterm infants.

## SUBJECTS AND METHODS

The Canadian Neonatal Network (CNN) collects data from 30 hospitals encompassing >90% of the level III NICU beds in Canada (32). Data were entered for infants admitted to NICUs by abstractors and submitted to the coordinating centre after local research ethics board or quality improvement committee approval (32). The details of data collection and management are published elsewhere (33) and the data were proven to be highly accurate (34). Research ethics board approval for this study was granted by St. Michael’s Hospital in Toronto, Ontario, Canada and the project was approved by the executive committee of the CNN.

Data of infants admitted from 2009 to 2014 that met the inclusion criteria of having a positive blood and/or cerebrospinal fluid culture within 3 days of birth were compared with the data from a previously published cohort of infants admitted from 2003 to 2008 (28). To compare patterns over time, the new cohort was divided into two groups: 2009 to 2011 and 2012 to 2014. These groups were also compared with two previously reported and published cohorts from 2003 to 2005 and 2006 to 2008. Exclusion criteria included a positive culture or detection by polymerase chain reaction (PCR) for viruses, fungi, or bacteria considered to be nonpathogenic, including coagulase-negative Staphylococci (CoNS). Cultures positive for multiple organisms were also excluded because of presumed contamination.

## STATISTICAL METHODS

Statistical analysis was conducted using SAS version 9.4 (Cary, NC). Descriptive statistics were used to summarize demographic data and organism distribution. To compare time periods, as well as preterm and term infants, two-tailed t-tests were used for continuous variables, and chi-square tests were used for categorical variables. Using a point from each year, linear best-fit lines were used to estimate trends in causative organisms for the 12-year cohort. The slopes of the best fit-lines were calculated using linear regression and were compared to a slope of zero to determine statistical significance.

## RESULTS

**Figure 1.**
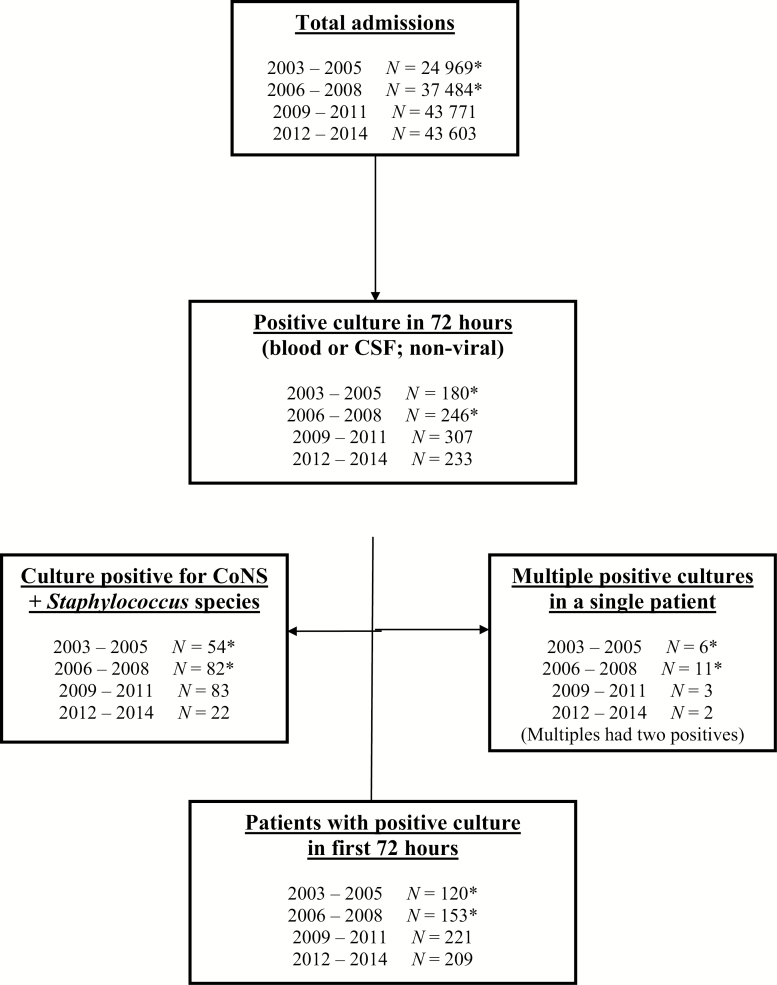
Flow diagram of selection of patients. Between 2009 and 2014, there were 87 374 NICU admissions, of which 430 infants met the inclusion criteria. Specifically, infants with positive blood and/or CSF cultures in the first 72 hours of birth were considered to have EONS. Infants with cultures positive for viruses, CoNS, other *Staphylococcus* species, or multiple organisms were excluded. The data shown reflect the process for case selection and exclusion. *For complete 2003–2008 values, refer to Sgro et al. 2011 (31). *CoNS Coagulase-negative Staphylococci; CSF Cerebrospinal fluid; EONS Early-onset neonatal sepsis; NICU Neonatal intensive care unit*.

Between 2009 and 2014, there were 87,374 NICU admissions, of which 430 infants (0.49%) developed EONS (Figure 1). The EONS incidence rates for all admissions between 2009 to 2011 and 2012 to 2014 were 0.50 and 0.48%, respectively.

Baseline demographic information for neonates born during the study period, 2009 to 2014, was similar for the two time periods analyzed: 2009 to 2011 and 2012 to 2014 (Table 1). *Escherichia coli* was the predominant organism in preterm infants, whereas GBS remained the most common pathogen in term infants. In preterm infants, defined as <37 weeks gestational age, *E coli*, GBS, and other organisms were causative of EONS in 2.58, 0.96, and 1.49 per 1,000 NICU admissions. When preterm infants, <29 weeks or less than 1,500 g were analyzed, *E coli* followed by GBS were the most common causes of EONS. Whereas in term infants, *E coli,* GBS, and other organisms were implicated in 1.70, 1.91, and 1.82 infants per 1,000 admissions (P<0.01). There were no cases of *Listeria monocytogenes* reported causing EONS.

**Table 1.** Baseline demographics of infants with EONS

		2009–2011	2012–2014	P
Number of patients with positive cultures		221	209	
Gestational age at birth (weeks)	Mean ± SD	31.2 ± 5.8	30.9 ± 5.6	0.53
	Median (range)	30 (23,45)	30 (23,42)	
Birth weight (grams)	Mean ± SD	1,894 ± 1,166	1,811 ± 1,102	0.45
	Median (range)	1,510 (433, 5,195)	1,460 (490, 4,552)	
Very-low birth weight (%)		108/221 (49%)	111/209 (53%)	0.38
<32 weeks (%)		121/221 (55%)	131/209 (63%)	0.24
32–37 weeks (%)		37/221 (17%)	30/209 (14%)	
>37 weeks (%)		63/221 (29%)	48/209 (23%)	
Male (%)		122/221 (55%)	122/209 (58%)	0.51
Apgar score at 5 minutes <7 (%)		90/221 (41%)	95/206 (46%)	0.26
Prolonged rupture of membrane (%)		91/144 (63%)	111/201 (55%)	0.14
Maternal chorioamnionitis (%)		80/181 (44%)	80/158 (51%)	0.24
Maternal antenatal steroid exposure (%)		130/218 (60%)	131/201 (65%)	0.24
Vaginal birth (%)		129/221 (58%)	110/209 (53%)	0.23
Meningitis (CSF) and Blood Culture Positive (%)		0/221 (0%)	7/209 (3%)	
Meningitis (%)		4/221 (2%)	21/209 (10%)	<0.01
Death within 7 days of positive culture (%)		9/221 (4%)	0	<0.01

*CSF Cerebrospinal fluid; EONS Early-onset neonatal sepsis.*

*For 2003–2005 and 2006–2008, refer to Sgro et al. 2011 (31).

**Number of infants admitted to all units during time period.

^†^Rate is relative to total admissions.

Very-low birth weight was defined as <1,500 g; Prolonged rupture of membrane was defined as >24 h before onset of labour.

**Figure 2.**
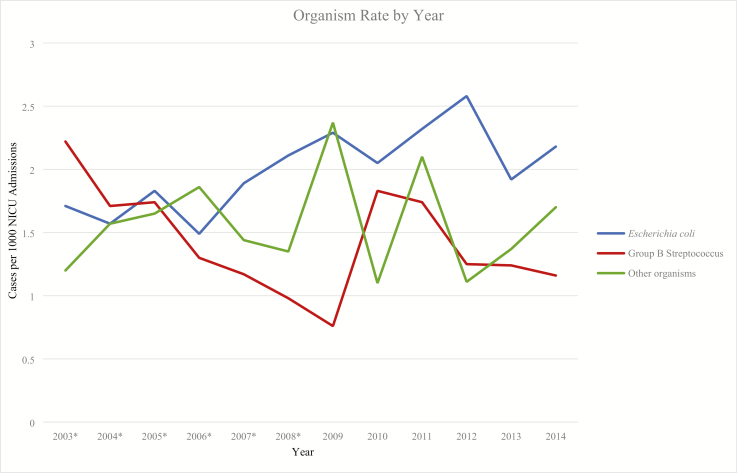
Yearly data on rates of EONS by organism. There was a significant increase in the number of EONS cases caused *E coli* from 2003 to 2014 (P<0.01) however, when analyzed from 2009 to 2014 there was no statistically significant change in the pattern of bacteria causing EONS. Data shown reflect the rates of EONS due to *E coli*, GBS, and other organisms over time. *For complete 2003–2008 values, refer to Sgro et al. 2011 (31). *EONS Early-onset neonatal sepsis; GBS Group B Streptococcus*.

Overall, the most commonly identified organism was *E coli*, followed by GBS (Table 2). Other common organisms included Streptococcus species and *Haemophilus influenzae.* There was no statistically significant change in the pattern of bacteria causing EONS between 2009 and 2014 however, when analyzed from 2003 to 2014 there was a significant increase in *E coli* EONS (P<0.01; Figure 2). In 2003, *E coli* rate was 1.71 infants per 1,000 admissions and in 2014, 2.18 infants per 1,000 admissions (Supplementary Appendix A, Tables 1and 2).

**Table 2.** Organism distribution according to gestational age at birth categories for positive blood or CSF cultures (%)

	2003–2005	2006–2008	2009–2011				2012–2014				2003–2014
Organisms	Total	Total	<32 weeks	32 - 37 weeks	>37 weeks	Total	<32 weeks	32 - 37 weeks	>37 weeks	Total	Total
*Escherichia coli*	33.9	40.9	49.2	43.2	20.6	40.2	53.0	63.3	16.3	46.0	41.0
GBS	36.4	25.2	18.5	21.6	44.4	26.3	19.7	13.3	46.9	25.1	27.4
Streptococcus Viridans Group	12.4	10.1	1.6	10.8	6.3	4.5	5.3	10.0	12.2	7.6	8.0
Other Streptococcus Species	5.0	5.0	7.3	18.9	12.7	10.7	0.8	3.3	12.2	3.8	6.4
*Haemphuilus Influenzae*	5.0	7.5	6.5	2.7	3.2	4.9	10.6	0.0	0.0	6.6	6.0
*Streptococcus Pneumoniae*	3.3	3.8	1.6	0.0	9.5	3.6	1.5	3.3	6.1	2.8	3.4
Enterococcus	0.8	1.3	0.8	2.7	1.6	1.3	3.8	0.0	4.1	3.3	1.8
*Klebsiella*	0.8	1.3	1.6	0.0	0.0	0.9	3.0	3.3	0.0	2.4	1.4
*Pseudomonas Aeruginosa* + Serratia	0.0	0.6	5.6	0.0	0.0	3.1	0.0	0.0	0.0	0.0	1.1
*Enterobacter*	0.8	1.9	0.0	0.0	0.0	0.0	0.0	0.0	0.0	0.0	0.6
Other	0.0	0.0	1.6	0.0	0.0	0.9	2.3	3.3	0.0	1.9	0.8
Total	100.0	100.0	100.0	100.0	100.0	100.0	100.0	100.0	100.0	100.0	100.0

*CSF Cerebrospinal fluid*; GBS Group B Streptococcus.

## DISCUSSION

In this study, we identified that the most common organism causing EONS in Canadian NICUs was *E coli*. There was no difference in the rate of EONS, demographic characteristics among neonates who developed EONS, or the organisms causing EONS between 2009 to 2011 and 2012 to 2014. Overall, there was an increase in *E coli* from 2003 to 2014; however, most of this increase occurred between 2003 and 2008 and no significant increase was identified from 2009 to 2014.

Our group previously reported 62,453 NICU admissions between 2003 and 2008, of which 273 infants met the inclusion criteria (Figure 1). Thus, over 12 years, there were 149,827 NICU admissions and 703 infants that met the inclusion criteria (Figure 1). When organism patterns were analyzed from 2003 to 2014, there was a significant increase in *E coli* EONS (P<0.01), and a decrease in GBS that was not statistically significant (P=0.13; Figure 2).

We report an EONS rate of 4.7/1,000 admissions, based on infants admitted to Canadian Level III NICUs in a 12-year cohort. With the exception of a study from the UK that included infants of all gestational ages and reported an EONS incidence of 9/1,000 admissions (6), NICU-based estimates focus predominantly on very low birth weight (VLBW) infants and report rates ranging from 9 to 26/1,000 admissions (7,14,15). Our reported incidence rate may be lower than in these previous reports because we included infants of all gestational ages.

We observed GBS more often in term infants, whereas *E coli* was the predominant pathogen in preterm infants (P<0.01). These findings are consistent with those reported in our previous study (31) as well as other international studies (2,3,13,14).

Our group previously found a decrease in GBS EONS between 2003 and 2008. However, once the analysis was expanded from 2003 to 2014, there was not a significant decrease in GBS EONS (P=0.13). A large NICU-based study in the USA identified a similar trend (13), and a US-based analysis of IAP also found a plateau in GBS EONS rates after the initial decline (35). In contrast to the decreasing GBS EONS trend, we report a significant trend of increasing *E coli* EONS between 2003 and 2014 (P<0.01). However, this trend plateaued from 2008 onwards. Our report is in discordance with some studies (14,26) and is concordant with other reports (12,13,25). Several groups have reported an association between IAP and ampicillin-resistant *E coli* EONS (4,17,26). Unfortunately, we have not collected information on drug resistance in our cohort; therefore, we cannot comment on this question.

When interpreting the trends in causative organisms presented, it is important to consider that VLBW and lower gestational age infants make up a greater proportion of the later cohorts. This likely suggests that the changing distribution of organisms is, at least partially, due to a shift in the mean gestational age of the cohort which may reflect changing referral patterns to Level III NICUs (27–29). For the previously published cohorts from 2003 to 2005 and 2006 to 2008 (31), mean gestational age at birth was 33.8 ± 5.5 and 32.4 ± 5.5 weeks, respectively. Likewise, mean birth weight was 2,361 ± 1,147 and 2,121 ± 1,172 g, respectively.

Baseline demographic characteristics of the cohorts were comparable between 2009 to 2011 and 2012 to 2014, with the exception of meningitis (P<0.01) and mortality (P<0.01) rates. However, these differences are of questionable clinical significance given that death and meningitis in EONS were uncommon throughout the study period. Recent literature reports EONS mortality rates ranging from 1.3 to 34.3%, with death being much more likely in preterm or VLBW infants (3,10,13–15). Our study design did not allow us to separate deaths attributable to EONS from deaths due to other factors. As a result, it is difficult to interpret this finding as it may reflect the overall decline in neonatal mortality attributable to advances in perinatal care (12,36). Our finding that meningitis remained uncommon corroborates other recent findings (2,13,37).

CoNS does not appear to be a major cause of EONS in the later cohort. In our previous report published on this cohort (31), the *Staphylococcus* species, including CoNS, were incorporated into organism trend calculations. From 2012 to 2014, only 22 cultures positive for Staphylococci were reported. Positive cultures in the context of these contaminants are widely reported (2,15,18), and our finding is likely due to shifts in medical practice and reporting due to clinicians recognizing these positive cultures as likely contaminants.

Our study has several unique strengths. We included infants of all gestational ages, thereby providing a complete picture of the burden of EONS in Canadian Level III NICUs. Additionally, we studied a large population of infants, capturing >90% of level III NICU admissions in Canada; thus, we presumably have data on the sickest infants with EONS.

There are limitations to our study. First, prior to 2009 the CNN database did not capture 100% of level III NICUs (32). Our study is population-based for neonates admitted to level III NICUs; however, there were likely some neonates admitted to level II NICUs when diagnosed with EONS who are not accounted for in our study. Thus, our reported rates may underestimate the true incidence of EONS. Second, limitations in data extraction did not allow us to determine if the cause of death in infants who did not survive was attributable to EONS or other causes such as congenital anomalies. Third, we were also unable to determine if positive cultures were evidence of true infection or contaminants. Next, the data were extracted from a national database and thus some information such as duration of ruptured membranes and administration of intrapartum antibiotic prophylaxis could not be obtained. Lastly, we have not captured the resistance pattern of causative bacteria. This is an important area for future study in order to develop appropriate guidelines for prophylaxis and treatment.

## CONCLUSIONS

The most common organism causing EONS in Canadian NICUs was *E coli*. There was no difference in the rate of EONS, demographic characteristics among neonates who developed EONS, and organisms causing EONS between 2009 to 2011 and 2012 to 2014. Overall, there was an increase noted in *E coli* from 2003 to 2014; however, most of this increase occurred from 2003 to 2008 and no significant increase was identified between 2009 and 2014.

Continued surveillance of causative organisms of EONS is imperative for accurate assessment and intervention and to allow for appropriate prevention strategies and effective empiric treatment regimens.

## Supplementary Material

pxz073_suppl_supplementary-Appendix_A

pxz073_suppl_supplementary-Appendix_B

## Notes

**Prior Presentation of Study Data**: Poster at the 2017 Canadian Paediatric Society Conference.
