# Supplementary material for: Early-onset neonatal sepsis: Organism patterns between 2009 and 2014
Source: Paediatr Child Health. 2019 Aug 9;25(7):425–31. doi: 10.1093/pch/pxz073 (PMC7606168; doi:10.1093/pch/pxz073)
Supplement: pxz073_suppl_supplementary-Appendix_A [file pxz073_suppl_supplementary-appendix_a.docx]

**Title:** Early Onset Neonatal Sepsis: Organism Patterns Between 2009 and 2014

**Appendix A: Organisms by Gestational Age and Year**

**Table 1.** Organisms by Gestational Age at Birth (2009-2014)

|  | **Number of Organisms per 1000 Patients** | | |
| --- | --- | --- | --- |
| **Organism** | **GA ≥ 37** | **GA< 37** | **p-value** |
| *Escherichia coli* | 1.70 | 2.58 | <0.01 |
| Group B Streptococcus | 1.91 | 0.96 |  |
| Total of other organisms | 1.82 | 1.49 |  |
| **Total rate** | **5.43** | **5.03** | **0.39** |

All numbers are number of cases per 1000 NICU admissions.

GA, gestational age.

**Table 2**. Yearly Data on Rates of EONS by Organism

|  | **2009** | **2010** | **2011** | **2012** | **2013** | **2014** | **p** | **Slope for trend** |
| --- | --- | --- | --- | --- | --- | --- | --- | --- |
| *Escherichia coli* | 2.29 | 2.05 | 2.32 | 2.58 | 1.92 | 2.18 | 0.75 | -0.021 |
| Group B Streptococcus | 0.76 | 1.83 | 1.74 | 1.25 | 1.24 | 1.16 | 0.94 | -0.009 |
| Other organisms | 2.37 | 1.10 | 2.10 | 1.11 | 1.37 | 1.70 | 0.49 | -0.101 |
| **Total** | 5.42 | 4.98 | 6.16 | 4.94 | 4.53 | 5.04 | 0.40 | -0.135 |

All numbers are number of cases per 1000 NICU admissions
