# Supplementary material for: Early-onset neonatal sepsis: Organism patterns between 2009 and 2014
Source: Paediatr Child Health. 2019 Aug 9;25(7):425–31. doi: 10.1093/pch/pxz073 (PMC7606168; doi:10.1093/pch/pxz073)
Supplement: pxz073_suppl_supplementary-Appendix_B [file pxz073_suppl_supplementary-appendix_b.docx]

**Canadian Neonatal Network Investigators**

Prakesh S Shah, MD, MSc (Director, Canadian Neonatal Network and site investigator), Mount Sinai Hospital, Toronto, Ontario; Jaideep Kanungo, MD, Victoria General Hospital, Victoria, British Columbia; Joseph Ting, MD, B.C. Women’s Hospital and Health Centre, Vancouver, British Columbia; Zenon Cieslak, MD, Royal Columbian Hospital, New Westminster, British Columbia; Rebecca Sherlock, MD, Surrey Memorial Hospital, Surrey, British Columbia; Wendy Yee, MD, Foothills Medical Centre, Calgary, Alberta; Jennifer Toye, MD, Royal Alexandra Hospital, Edmonton, Alberta; Carlos Fajardo, MD, Alberta Children’s Hospital, Calgary, Alberta; Zarin Kalapesi, MD, Regina General Hospital, Regina, Saskatchewan; Koravangattu Sankaran, MD, MBBS, and Sibasis Daspal, MD, Royal University Hospital, Saskatoon, Saskatchewan; Mary Seshia, MBChB, Winnipeg Health Sciences Centre, Winnipeg, Manitoba; Ruben Alvaro, MD, St. Boniface General Hospital, Winnipeg, Manitoba; Amit Mukerji, MD, Hamilton Health Sciences Centre, Hamilton, Ontario; Orlando Da Silva, MD, MSc, London Health Sciences Centre, London, Ontario; Chuks Nwaesei, MD, Windsor Regional Hospital, Windsor, Ontario; Kyong-Soon Lee, MD, MSc, Hospital for Sick Children, Toronto, Ontario; Michael Dunn, MD, Sunnybrook Health Sciences Centre, Toronto, Ontario; Brigitte Lemyre, MD, Children’s Hospital of Eastern Ontario and Ottawa General Hospital, Ottawa, Ontario; Kimberly Dow, MD, Kingston General Hospital, Kingston, Ontario; Ermelinda Pelausa, MD, Jewish General Hospital, Montréal, Québec; Keith Barrington, MBChB, and Anie Lapoint, MD, Hôpital Sainte-Justine, Montréal, Québec; Christine Drolet, MD, and Bruno Piedboeuf, MD, Centre Hospitalier Universitaire de Québec, Sainte Foy, Québec; Martine Claveau, MSc, LLM, NNP, and Marc Beltempo, MD, McGill University Health Centre, Montréal, Québec; Valerie Bertelle, MD, and Edith Masse, MD, Centre Hospitalier Universitaire de Sherbrooke, Sherbrooke, Québec; Roderick Canning, MD, Moncton Hospital, Moncton, New Brunswick; Hala Makary, MD, Dr. Everett Chalmers Hospital, Fredericton, New Brunswick; Cecil Ojah, MBBS, and Luis Monterrosa, MD, Saint John Regional Hospital, Saint John, New Brunswick; Akhil Deshpandey, MBBS, MRCPI, Janeway Children’s Health and Rehabilitation Centre, St. John’s, Newfoundland; Jehier Afifi, MB BCh, MSc, IWK Health Centre, Halifax, Nova Scotia; Andrzej Kajetanowicz, MD, Cape Breton Regional Hospital, Sydney, Nova Scotia; Shoo K Lee, MBBS, PhD (Chairman, Canadian Neonatal Network), Mount Sinai Hospital, Toronto, Ontario.
